# Endometroid carcinoma developing in endometriosis over the symphysis pubis^☆^

**DOI:** 10.1016/j.gynor.2013.08.001

**Published:** 2013-09-10

**Authors:** Michaela Peer, Wolfgang Fellner, Beata E. Seeber, Alain G. Zeimet, Christian Marth

**Affiliations:** a Department of Obstetrics and Gynecology, Innsbruck Medical University, Innsbruck, Austria; b Department of Obstetrics and Gynecology, Vöcklabruck General Hospital, Vöcklabruck, Austria; c Department of Endocrinology and Reproductive Medicine, Innsbruck Medical University, Innsbruck, Austria

**Keywords:** Endometriosis, Endometrial cancer, Malignant transformation

## Abstract

• We present the case of a premenopausal woman, who developed an endometriosis based endometrial carcinoma at the mons pubis.
• After a three year follow-up period the patient remains disease free.

## Case report

**Fig. 1:**
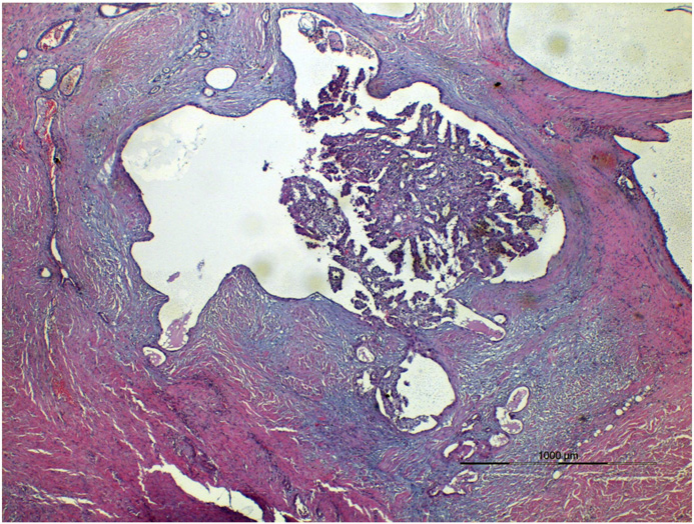
Endometroid CA within endometriotic cyst.

We here present the case of a 44-year-old nullipara (BMI 28.7 kg/m^2^) with no significant past gynecological, medical or surgical history other than an open appendectomy. She presented to the general surgeons with a subcutaneous lump on the mons pubis that had been monitored for about one year. MRI showed a polycystic mass of 30 mm × 18 mm × 18 mm size that was excised and described as an adenocarcinoma on the basis of endometriosis (Fig. 1).

Gynecological examination and transvaginal pelvic ultrasound were normal. The patient had an intra-uterine device (IUD) in-situ, which had been placed for contraceptive purposes only. PAP smear and tumor markers (CEA, AFP, CA 12-5, CA 19-9, CA 15-3, SCC) were all normal. Repeat MRI showed partly solid, partly cystic post-operative residuals in the right inguinal region, extending up to the right edge of the symphysis pubis with uncertain boney infiltration. A PET and a CT scan revealed pathological lymph nodes on the right and one lesion in the left inguinal region. The patient was transferred to our Department for tertiary care. Following discussion by the Gynecologic Cancer Inter-Group (GCIC), the residual tumor was removed one month after the initial surgery. This involved a multidisciplinary team of gynecologic, orthopedic and plastic surgeons and included the resection of the mons pubis and the right pelvic and inguinofemoral lymph nodes. The symphysis pubis was partly removed and the right pectineus muscle was resected; the rectus sheath and the rectus muscle were refixated onto the remainder of the symphysis pubis. The skin of the left lower abdomen was transposed to cover the defect. A laparoscopic tubal ligation, removal of the LNG-IUS and dilatation and curettage (D&C) were also performed. During laparoscopy no evidence of endometriosis was seen; the D&C demonstrated a normal endometrial mucosa without atypia.

The histology report revealed large areas of highly differentiated endometrioid adenocarcinoma and small areas of adeno-squamous as well as serous-papillary differentiation. The tumor margins were free. Furthermore, areas of endometriosis without atypia were seen. All 12 lymph nodes resected were tumor-free. Immunohistochemistry confirmed the presence of estrogen and progesterone receptors, as well as expression of the L1 cell adhesion molecule (L1CAM) in the endometriotic and the tumor tissue.

Following a further discussion by the Gynecologic Cancer Inter-Group (GCIC) the patient was to receive six cycles of adjuvant chemotherapy with carboplatin/paclitaxel and local radiotherapy. During the first cycle of chemotherapy the patient developed a major anaphylactic reaction. She also reacted to the alternative option of pegylated liposomal doxorubicin and docetaxel. Finally a combination of epirubicin and carboplatin was tolerated well and the remaining cycles could be administered without unexpected toxicities. The patient received local radiotherapy with 5000 cGy in 25 fractions to the area of the previous malignancy as well as up to 6000 cGy in five further fractions to the whole operating field. She was followed up clinically and paraclinically for the last three years and remains well with no evidence of tumor recurrence.

## Discussion

The current literature includes one case of inguinal endometriosis (Jubanyik and Comite, 1997). However, to our knowledge this is the first case documenting ectopic endometrium in the mons pubis.

Endometriosis usually affects women of reproductive age and has a prevalence of 10% (Wheeler, 1989). Its etiology is controversial, with common opinion being that endometriosis develops via retrograde menstruation, whereby the uterine endometrial glands and stroma mainly implant onto surfaces and organs of the pelvis (Jubanyik and Comite, 1997).

In the malignant transformation of endometriosis the ovary is the primary site in 79% of all cases (Hoang et al., 2005). Extragonadal sites, e.g. the rectovaginal septum, pelvic peritoneum or the abdominal wall, are identified in 21% (Hoang et al., 2005). The malignant transformation of endometriosis is rare and the case presented here is certainly highly unusual with a novel location of endometriosis and its malignant transformation in a pre-menopausal woman. The histology report revealed features of Type I and Type II endometrial cancer, with highly differentiated adenocarcinoma having developed next to serous-papillary differentiated tissue. Both cancerous and endometriotic tissues were positive for L1CAM-antibodies, which are associated with a high risk of tumor relapse and short survival rates (Zeimet et al., 2013). Nevertheless, after a three year follow-up there is no evidence of disease recurrence.

In this case the possibility of metastasis from a uterine primary was excluded. Nevertheless, we cannot exclude the possibility of endometriosis implantation at the time of an earlier appendectomy. The surgical incision was thereby made in the right lower quadrant. An oophorectomy at the time of surgical resection was not considered in this patient, however, it could certainly be deliberated in future cases given that the ovary is the most common site of endometriosis-associated adenocarcinoma. There are multiple possible explanations for the pre-malignant potential of endometriosis. Pollacco et al., for example, propose an endometriosis-induced carcinoma (EIC) triad, with endocrinological and immunological factors as well as genomic alterations leading to the malignant transformation of the endometriotic tissue (Pollacco et al., 2012). Certainly, more research needs to be conducted to analyze the possible mechanisms of cancer formation from endometriosis.

## Conflict of interest

The authors report no conflict of interest.
